# Supplementary material for: MicroRNA characteristics in epithelial ovarian cancer
Source: PLoS One. 2021 Jun 4;16(6):e0252401. doi: 10.1371/journal.pone.0252401 (PMC8177468; doi:10.1371/journal.pone.0252401)
Supplement: S1 File — (DOCX) [file pone.0252401.s001.docx]

**S1 File. Supplementary Methods**

**Validation cohorts**

Three external datasets (GSE25204, GSE73582, GSE73581) were retrieved from the NCBI Gene Expression Omnibus database (1). All datasets have been used for investigation and validation in previous reports where they are described in further details (2-4). Two of the datasets; GSE25204 and GSE73582, including 130 patients with FIGO stage I-IV and 133 patients with FIGO stage III-IV respectively, were combined and treated as one dataset with a total of 263 patients with EOC (2, 3). MiRNA profiles for the cohorts GSE25204 and GSE73582 were analyzed from either FFPE or frozen samples and profiled with Illumina microchip platform (Illumina, San Diego, CA, USA). The third cohort; GSE73581 included a total of 179 EOC patients with FIGO stage I-IV, miRNA profiles for this cohort was profiled from FFPE tissue samples with Agilent miRNA microarray platform (Agilent Technologies, Santa Clara, CA, USA) (2, 4).

Clinical information on FIGO stage and residual tumor after primary surgery were defined in the same manner in the external cohorts and in our cohort. As for histologic subtype and tumor grade there were some slight differences. Patients who did not meet our inclusion criteria for histologic subtype (39 patients from the GSE25204+GSE73582 cohort and 25 patients from the GSE73581) and tumor grade (28 patients from the GSE25204+GSE73582 cohort and 21 patients from the GSE73581 cohort) were excluded in the analysis of histologic subtype and tumor grade respectively (Supplementary table1). Type I and type II OC tumors were not evaluated in the external cohorts due to differences in definition of histologic subtypes and tumor grade.

1. Clough E, Barrett T. The Gene Expression Omnibus Database. Methods in molecular biology (Clifton, NJ). 2016;1418:93-110.

2. Bagnoli M, Canevari S, Califano D, Losito S, Maio MD, Raspagliesi F, et al. Development and validation of a microRNA-based signature (MiROvaR) to predict early relapse or progression of epithelial ovarian cancer: a cohort study. The Lancet Oncology. 2016;17(8):1137-46.

3. Bagnoli M, De Cecco L, Granata A, Nicoletti R, Marchesi E, Alberti P, et al. Identification of a chrXq27.3 microRNA cluster associated with early relapse in advanced stage ovarian cancer patients. Oncotarget. 2011;2(12):1265-78.

4. Pignata S, Scambia G, Ferrandina G, Savarese A, Sorio R, Breda E, et al. Carboplatin plus paclitaxel versus carboplatin plus pegylated liposomal doxorubicin as first-line treatment for patients with ovarian cancer: the MITO-2 randomized phase III trial. Journal of clinical oncology : official journal of the American Society of Clinical Oncology. 2011;29(27):3628-35.
