# Supplementary material for: MicroRNA characteristics in epithelial ovarian cancer
Source: PLoS One. 2021 Jun 4;16(6):e0252401. doi: 10.1371/journal.pone.0252401 (PMC8177468; doi:10.1371/journal.pone.0252401)
Supplement: S1 Table — (DOCX) [file pone.0252401.s002.docx]

| **S1 Table. Baseline characteristics of the validation cohorts** | | |
| --- | --- | --- |
|  | **GSE25204 + GSE73582**  **n = 263** | **GSE73581**  **n = 179** |
| Median age in year (range) | 55 (25-85) | 58 (28-78) |
| Histology |  |  |
| Serous adenocarcinoma | 190 (72%) | 124 (69%) |
| Mucinous adenocarcinoma | 1 (<1%) | - |
| Endometrioid adenocarcinoma | 26 (10%) | 24 (13%) |
| Clear Cell adenocarcinoma | 7 (3%) | 6 (3%) |
| Undifferentiated | 23 (9%) | 10 (6%) |
| Others and mixes | 15 (6%) | 13 (7%) |
| Missing information | 1 (<1%) | 2 (1%) |
| FIGO stage |  |  |
| I | 16 (6%) | 17 (9%) |
| II | 9 (3%) | 15 (8%) |
| III | 212 (81%) | 123 (69%) |
| IV | 26 (10%) | 24 (13%) |
| Histologic grade |  |  |
| 1 | 7 (3%) | 5 (3%) |
| 2 | 51 (19%) | 27 (15%) |
| 3 | 177 (67%) | 126 (70%) |
| Unknown | 2 (1%) | 11 (6%) |
| Borderline | 3 (1%) | - |
| Undifferentiated | 23 (9%) | 10 (6%) |
| Residual tumor after surgery |  |  |
| 0 (radical surgery) | 76 (29%) | 73(41%) |
| <1 cm | 85 (32%) | 42 (23%) |
| ≥1cm | 101 (38%) | 53 (30%) |
| Not operated | - | 11 (6%) |
| Missing information | 1 (<1%) | - |
| Chemotherapy |  |  |
| Carboplatin + Paclitaxel |  | 78 (44%) |
| Carboplatin - Caelyx |  | 101 (56%) |
| Median follow-up (months) | 44 (24-71) | 73 (60-88) |
| OS = overall survival  FIGO = International Federation of Gynecology and Obstetrics | | |
