# Supplementary material for: MicroRNA characteristics in epithelial ovarian cancer
Source: PLoS One. 2021 Jun 4;16(6):e0252401. doi: 10.1371/journal.pone.0252401 (PMC8177468; doi:10.1371/journal.pone.0252401)
Supplement: S2 Table — (DOCX) [file pone.0252401.s003.docx]

| **S2 Table. Univariate logistic regression analysis of miRNAs associated with clinicopathologic characteristics** | | | |
| --- | --- | --- | --- |
|  | **OR** | **95% CI** | **P-value** |
| **Histology (serous vs. others)** | | | |
| **miR-92a^1^** | 0.11 | 0.05 - 0.27 | <.0001 |
| **let-7e** | 0.14 | 0.05 - 0.35 | <.0001 |
| **miR-130a** | 0.21 | 0.11 - 0.37 | <.0001 |
| **miR-125a-5p** | 0.23 | 0.12 - 0.46 | <.0001 |
| **miR-23a** | 0.24 | 0.11 - 0.52 | 0.0003 |
| **miR-125a-3p** | 0.29 | 0.16 - 0.53 | <.0001 |
| **miR-146b-3p** | 0.33 | 0.17 - 0.62 | 0.0006 |
| **miR-27a*** | 0.33 | 0.18 - 0.59 | 0.0002 |
| **miR-106b*** | 0.34 | 0.19 - 0.60 | 0.0002 |
| **miR-17^1^** | 0.34 | 0.19 - 0.62 | 0.0004 |
| **miR-378** | 0.34 | 0.22 - 0.53 | <.0001 |
| **miR-106a** | 0.35 | 0.19 - 0.62 | 0.0003 |
| **miR-193a-5p** | 0.35 | 0.21 - 0.57 | <.0001 |
| **miR-378*** | 0.35 | 0.23 - 0.52 | <.0001 |
| **miR-146b-5p** | 0.36 | 0.25 - 0.53 | <.0001 |
| **miR-20a** | 0.36 | 0.22 - 0.61 | <.0001 |
| **miR-30c-1*** | 0.36 | 0.21 - 0.64 | 0.0005 |
| **miR-193a-3p** | 0.37 | 0.25 - 0.55 | <.0001 |
| **miR-25** | 0.37 | 0.22 - 0.62 | 0.0002 |
| **miR-422a** | 0.37 | 0.25 - 0.55 | <.0001 |
| **miR-92b** | 0.40 | 0.27 - 0.60 | <.0001 |
| **miR-17*^1^** | 0.47 | 0.33 - 0.67 | <.0001 |
| **miR-224** | 0.47 | 0.31 - 0.71 | 0.0003 |
| **miR-149** | 0.48 | 0.36 - 0.65 | <.0001 |
| **miR-15b** | 0.48 | 0.32 - 0.74 | 0.0007 |
| **miR-181d** | 0.48 | 0.34 - 0.69 | <.0001 |
| **miR-24-2*** | 0.50 | 0.34 - 0.73 | 0.0003 |
| **miR-93*** | 0.50 | 0.35 - 0.73 | 0.0003 |
| **miR-30e** | 0.53 | 0.37 - 0.75 | 0.0005 |
| **miR-128** | 0.54 | 0.38 - 0.77 | 0.0007 |
| **miR-20b** | 0.54 | 0.39 - 0.75 | 0.0002 |
| **miR-19b^1^** | 0.60 | 0.45 - 0.80 | 0.0005 |
| **miR-18a^1^** | 0.61 | 0.47 - 0.80 | 0.0003 |
| **miR-187** | 0.73 | 0.61 - 0.88 | 0.001 |
| **miR-205** | 0.82 | 0.73 - 0.92 | 0.0009 |
| **miR-192** | 1.39 | 1.17 - 1.65 | 0.0002 |
| **miR-509-3p** | 1.40 | 1.21 - 1.62 | <.0001 |
| **miR-194** | 1.42 | 1.18 - 1.72 | 0.0002 |
| **miR-509-3-5p** | 1.42 | 1.21 - 1.66 | <.0001 |
| **miR-184** | 1.43 | 1.17 - 1.76 | 0.0006 |
| **miR-508-5p** | 1.48 | 1.24 - 1.77 | <.0001 |
| **miR-202** | 1.56 | 1.24 - 1.96 | 0.0002 |
| **miR-509-5p** | 1.56 | 1.26 - 1.93 | <.0001 |
| **miR-513c** | 1.57 | 1.28 - 1.93 | <.0001 |
| **miR-506** | 1.58 | 1.28 - 1.94 | <.0001 |
| **miR-513a-5p** | 1.63 | 1.29 - 2.07 | <.0001 |
| **miR-510** | 1.74 | 1.37 - 2.20 | <.0001 |
| **miR-513b** | 1.78 | 1.33 - 2.40 | 0.0001 |
| **miR-375** | 1.79 | 1.48 - 2.18 | <.0001 |
| **miR-409-3p** | 1.95 | 1.35 - 2.82 | 0.0004 |
| **miR-485-5p** | 1.96 | 1.37 - 2.82 | 0.0003 |
| **miR-411** | 2.10 | 1.38 - 3.20 | 0.0005 |
| **miR-134** | 2.11 | 1.47 - 3.03 | <.0001 |
| **miR-409-5p** | 2.18 | 1.44 - 3.30 | 0.0002 |
| **miR-214** | 2.48 | 1.50 - 4.11 | 0.0004 |
| **miR-370** | 2.67 | 1.72 - 4.14 | <.0001 |
| **miR-377*** | 2.75 | 1.53 - 4.95 | 0.0007 |
| **miR-192*** | 2.85 | 1.68 - 4.84 | 0.0001 |
| **miR-34a** | 3.13 | 1.77 - 5.55 | <.0001 |
| **miR-215** | 3.26 | 1.64 - 6.44 | 0.0007 |
| **miR-194*** | 3.40 | 1.92 - 6.01 | <.0001 |
| **miR-455-3p** | 6.58 | 3.06 - 14.15 | <.0001 |
| **miR-516a-3p** | 13.06 | 3.30 - 51.69 | 0.0003 |
| **miR-92a^1^** | 0.11 | 0.05 - 0.27 | <.0001 |
| **FIGO Stage (III+IV vs I+II)** | | | |
| **miR-506** | 0.69 | 0.57 - 0.83 | 0.0001 |
| **miR-375** | 0.76 | 0.66 - 0.88 | 0.0003 |
| **miR-508-5p** | 0.78 | 0.67 - 0.90 | 0.0009 |
| **miR-483-5p** | 1.53 | 1.20 - 1.93 | 0.0005 |
| **miR-146b-5p** | 1.68 | 1.30 - 2.16 | <.0001 |
| **miR-130a** | 1.93 | 1.33 - 2.81 | 0.0006 |
| **miR-23a** | 3.49 | 1.75 - 6.95 | 0.0004 |
| **let-7e** | 3.62 | 1.72 - 7.60 | 0.0007 |
| **miR-595** | 6.66 | 2.52 - 17.61 | 0.0001 |
| **Grade (2+3 vs. 1)** | | | |
| **miR-34a** | 0.08 | 0.03 - 0.23 | <.0001 |
| **miR-768-3p** | 0.21 | 0.09 - 0.50 | 0.0005 |
| **miR-181a** | 0.22 | 0.10 - 0.47 | <.0001 |
| **miR-134** | 0.40 | 0.24 - 0.64 | 0.0002 |
| **miR-127-3p** | 0.41 | 0.24 - 0.69 | 0.0009 |
| **miR-194*** | 0.52 | 0.37 - 0.73 | 0.0001 |
| **miR-184** | 0.53 | 0.41 - 0.69 | <.0001 |
| **miR-192*** | 0.54 | 0.38 - 0.75 | 0.0003 |
| **miR-375** | 0.56 | 0.46 - 0.69 | <.0001 |
| **miR-215** | 0.58 | 0.44 - 0.76 | <.0001 |
| **miR-422a** | 1.86 | 1.29 - 2.68 | 0.001 |
| **miR-149** | 1.90 | 1.37 - 2.63 | 0.0001 |
| **miR-18a^1^** | 2.07 | 1.46 - 2.94 | <.0001 |
| **miR-20b** | 2.12 | 1.43 - 3.14 | 0.0002 |
| **miR-146b-5p** | 2.19 | 1.55 - 3.09 | <.0001 |
| **miR-1301** | 2.67 | 1.60 - 4.46 | 0.0002 |
| **miR-18b** | 3.36 | 1.66 - 6.80 | 0.0008 |
| **Type I or II (II vs. I)** | | | |
| **hsa-miR-516a-3p** | 0.09 | 0.02 - 0.35 | 0.0004 |
| **hsa-miR-455-3p** | 0.19 | 0.1 - 0.38 | <.0001 |
| **hsa-miR-619** | 0.22 | 0.09 - 0.53 | 0.0007 |
| **hsa-miR-34a** | 0.26 | 0.14 - 0.47 | <.0001 |
| **hsa-miR-377*** | 0.32 | 0.18 - 0.57 | 0.0001 |
| **hsa-miR-215** | 0.34 | 0.18 - 0.64 | 0.0009 |
| **hsa-miR-194*** | 0.36 | 0.22 - 0.58 | <.0001 |
| **hsa-miR-192*** | 0.38 | 0.23 - 0.63 | 0.0001 |
| **hsa-miR-34a*** | 0.38 | 0.22 - 0.64 | 0.0003 |
| **hsa-miR-370** | 0.44 | 0.3 - 0.66 | <.0001 |
| **hsa-miR-409-5p** | 0.47 | 0.31 - 0.7 | 0.0002 |
| **hsa-miR-134** | 0.51 | 0.36 - 0.71 | <.0001 |
| **hsa-miR-409-3p** | 0.54 | 0.38 - 0.77 | 0.0006 |
| **hsa-miR-485-5p** | 0.55 | 0.39 - 0.77 | 0.0006 |
| **hsa-miR-513b** | 0.57 | 0.43 - 0.76 | 0.0001 |
| **hsa-miR-510** | 0.58 | 0.46 - 0.72 | <.0001 |
| **hsa-miR-375** | 0.59 | 0.49 - 0.71 | <.0001 |
| **hsa-miR-508-3p** | 0.61 | 0.46 - 0.81 | 0.0005 |
| **hsa-miR-509-5p** | 0.61 | 0.49 - 0.75 | <.0001 |
| **hsa-miR-513a-5p** | 0.61 | 0.49 - 0.77 | <.0001 |
| **hsa-miR-506** | 0.62 | 0.5 - 0.76 | <.0001 |
| **hsa-miR-513c** | 0.62 | 0.51 - 0.76 | <.0001 |
| **hsa-miR-184** | 0.64 | 0.52 - 0.79 | <.0001 |
| **hsa-miR-202** | 0.64 | 0.52 - 0.8 | <.0001 |
| **hsa-miR-508-5p** | 0.66 | 0.56 - 0.79 | <.0001 |
| **hsa-miR-509-3-5p** | 0.7 | 0.6 - 0.81 | <.0001 |
| **hsa-miR-509-3p** | 0.72 | 0.63 - 0.83 | <.0001 |
| **hsa-miR-18a^1^** | 1.72 | 1.31 - 2.24 | <.0001 |
| **hsa-miR-128** | 1.81 | 1.29 - 2.56 | 0.0007 |
| **hsa-miR-30e** | 1.81 | 1.29 - 2.54 | 0.0007 |
| **hsa-miR-20b** | 1.88 | 1.37 - 2.58 | <.0001 |
| **hsa-miR-17*** | 1.91 | 1.35 - 2.69 | 0.0002 |
| **hsa-miR-24-2*** | 1.96 | 1.36 - 2.82 | 0.0003 |
| **hsa-miR-181d** | 1.98 | 1.4 - 2.79 | 0.0001 |
| **hsa-miR-149** | 2.03 | 1.54 - 2.68 | <.0001 |
| **hsa-miR-224** | 2.03 | 1.39 - 2.97 | 0.0002 |
| **hsa-miR-1301** | 2.06 | 1.42 - 3.01 | 0.0002 |
| **hsa-miR-15b** | 2.07 | 1.37 - 3.14 | 0.0006 |
| **hsa-miR-92b** | 2.08 | 1.47 - 2.94 | <.0001 |
| **hsa-miR-193a-3p** | 2.14 | 1.52 - 3.03 | <.0001 |
| **hsa-miR-877** | 2.15 | 1.37 - 3.36 | 0.0008 |
| **hsa-miR-378*** | 2.17 | 1.55 - 3.05 | <.0001 |
| **hsa-miR-422a** | 2.29 | 1.61 - 3.26 | <.0001 |
| **hsa-miR-146b-5p** | 2.51 | 1.77 - 3.54 | <.0001 |
| **hsa-miR-378** | 2.51 | 1.69 - 3.73 | <.0001 |
| **hsa-miR-20a^1^** | 2.54 | 1.57 - 4.09 | 0.0001 |
| **hsa-miR-30c-1*** | 2.58 | 1.51 - 4.41 | 0.0005 |
| **hsa-miR-27a*** | 2.6 | 1.55 - 4.38 | 0.0003 |
| **hsa-miR-193a-5p** | 2.61 | 1.63 - 4.18 | <.0001 |
| **hsa-miR-760** | 2.64 | 1.48 - 4.7 | 0.001 |
| **hsa-miR-25** | 2.69 | 1.62 - 4.46 | 0.0001 |
| **hsa-miR-17^1^** | 2.81 | 1.6 - 4.96 | 0.0003 |
| **hsa-miR-106a** | 2.88 | 1.64 - 5.03 | 0.0002 |
| **hsa-miR-106b*** | 2.94 | 1.69 - 5.11 | 0.0001 |
| **hsa-miR-99b*** | 3.07 | 1.8 - 5.23 | <.0001 |
| **hsa-miR-125a-3p** | 3.68 | 2.02 - 6.7 | <.0001 |
| **hsa-miR-125a-5p** | 4.05 | 2.13 - 7.68 | <.0001 |
| **hsa-miR-23a** | 4.16 | 1.95 - 8.91 | 0.0002 |
| **hsa-miR-130a** | 4.45 | 2.53 - 7.82 | <.0001 |
| **hsa-miR-92a^1^** | 6.17 | 2.79 - 13.64 | <.0001 |
| **hsa-let-7e** | 9.31 | 3.6 - 24.11 | <.0001 |
| **Residual disease (radical vs. non-radical)** | | | |
| **miR-939** | 0.32 | 0.17 - 0.59 | 0.0003 |
| OR = odds ratio. CI= confidence interval  ^1^Member of the Oncomir-1 family (miR-17-92 cluster). | | | |
