# Supplementary material for: MicroRNA characteristics in epithelial ovarian cancer
Source: PLoS One. 2021 Jun 4;16(6):e0252401. doi: 10.1371/journal.pone.0252401 (PMC8177468; doi:10.1371/journal.pone.0252401)
Supplement: S3 Table — (DOCX) [file pone.0252401.s004.docx]

| **S3 Table. Combinations of miRNAs demonstrating a correlation greater than 0.9.** | | |
| --- | --- | --- |
| **mirna_1** | **mirna_2** | **r** |
| hsa-miR-506_st | hsa-miR-513c_st | 0.90013 |
| hsa-miR-34c-3p_st | hsa-miR-34c-5p_st | 0.90171 |
| hsa-miR-501-3p_st | hsa-miR-502-3p_st | 0.90217 |
| hsa-miR-506_st | hsa-miR-509-5p_st | 0.90502 |
| hsa-miR-127-3p_st | hsa-miR-134_st | 0.90686 |
| hsa-miR-195_st | hsa-miR-497_st | 0.91248 |
| hsa-miR-509-3-5p_st | hsa-miR-509-3p_st | 0.91696 |
| hsa-miR-500-star_st | hsa-miR-501-3p_st | 0.91752 |
| hsa-miR-181a_st | hsa-miR-181b_st | 0.92079 |
| hsa-miR-192_st | hsa-miR-194_st | 0.92745 |
| hsa-miR-362-5p_st | hsa-miR-532-5p_st | 0.93282 |
| hsa-miR-508-5p_st | hsa-miR-509-3-5p_st | 0.93656 |
| hsa-miR-221_st | hsa-miR-222_st | 0.93984 |
| hsa-miR-320a_st | hsa-miR-320c_st | 0.94482 |
| hsa-miR-103_st | hsa-miR-107_st | 0.95177 |
| hsa-miR-320b_st | hsa-miR-320c_st | 0.95769 |
| hsa-miR-500-star_st | hsa-miR-502-3p_st | 0.96412 |
| hsa-miR-449a_st | hsa-miR-449b_st | 0.97372 |
| hsa-miR-320a_st | hsa-miR-320b_st | 0.97443 |
| hsa-miR-199a-3p_st | hsa-miR-199b-3p_st | 0.97754 |
| hsa-miR-106a_st | hsa-miR-17_st | 0.99382 |
| r = correlation coefficient. | | |
